# Performance of Concentrically Loaded RC Wall-like Columns Upgraded with Innovative Hybrid NSM/CFRP System

**DOI:** 10.3390/polym15020378

**Published:** 2023-01-10

**Authors:** Hussein Elsanadedy, Husain Abbas, Tarek Almusallam, Yousef Al-Salloum

**Affiliations:** Chair of Research and Studies in Strengthening and Rehabilitation of Structures, Department of Civil Engineering, King Saud University, Riyadh 11421, Saudi Arabia

**Keywords:** wall-like RC columns, strengthening, FRP jacket, NSM rebars, testing, FE modeling

## Abstract

In RC (reinforced concrete) frame structures, wall-like columns are laid within the space occupied by masonry walls to maximize usable space and thus minimize the column projections into the usable area. These columns often require strengthening owing to various reasons, including increasing the number of stories, changes in building usage, and others. The use of a hybrid system comprising NSM (near-surface mounted) steel rebars combined with CFRP (carbon-fiber reinforced polymer) laminates may be considered a sound technique for strengthening such wall-like building columns. The prime aim of this study is to devise an efficient scheme using a hybrid NSM/CFRP system to strengthen existing RC wall-like columns. Six half-scale RC wall-like columns were prepared and tested under monotonic concentric axial compression. Two columns were unstrengthened to serve as control specimens (CW1 and CW2), and four specimens were strengthened using four different schemes (SW1, SW2, SW3, and SW4). As favored by architects, all strengthening schemes were designed so that the dimensions of the column cross-section were not increased. The effects of strengthening schemes on the enhancement of axial capacity, energy dissipated, and stiffness were evaluated to find the most efficient scheme. Among the four studied schemes, using vertical continuous NSM rebars in combination with the wrapping of the three CFRP layers onto the exterior column surface (in specimen SW2) was the most efficient as it enhanced the ultimate load capacity by 80%. Three-dimensional FE (finite element) analysis was also conducted to predict the response of test specimens. The test results matched well with the FE outputs, which justified the accuracy of the used constitutive models for concrete, steel rebars, and CFRP sheets.

## 1. Introduction

In order to maximize the space in RC buildings, wall-like columns are used in the space occupied by masonry walls. Such wall-like columns are extensively used in congested and expensive areas of metropolitan cities. However, throughout the Kingdom of Saudi Arabia, there are a number of wall-like RC columns that are exceptionally old, deteriorating, or carrying higher loads than those for which they were designed due to various reasons. Therefore, these columns need to be strengthened.

Until recently, the two most common methods for strengthening a deficient RC column were (i) constructing an additional RC jacket [1,2] and (ii) installing grout-injected steel jackets [3,4,5,6,7]. These methods are labor-intensive and occasionally difficult to implement on-site. In addition to being heavy, steel jackets are also poor in resisting weather. In recent years, strengthening RC columns using FRP composites has gained wide popularity and replaced steel jacketing substantially. The use of FRP composites is a sound technique for strengthening such wall-like building columns because composites ensure increased strength and/or ductility, fast and easy installation, high durability, low interruption to the users of the structure, and an almost negligible increase in the mass and geometrical dimensions of the column cross-sections.

There are several studies dealing with the numerical modeling of FRP-wrapped members, such as (i) circular RC columns wrapped with partial FRP strips [8], (ii) FRP retrofitted members having deficient lap splices [9,10,11], (iii) rectangular RC columns strengthened by FRP wrapping under axial [12] and cyclic axial compression [13], and (iv) general cases of confined concrete [14] using Drucker–Prager plasticity models [15]. The axial behavior of FRP sections was also studied numerically [16]. Other techniques of retrofitting have also been investigated, such as the use of precast fiber-reinforced cementitious composites [17].

Investigations of the compressive behavior of RC columns of rectangular sections wrapped with epoxy-bonded FRP sheets have mostly studied the effect of the aspect ratio of column sections varying from 1 to 2. Some studies [18,19,20,21,22,23,24,25,26] involving wall-like columns were conducted. Tan [18] tested wall-like columns under axial load to study the effect of the type of fibers, their configuration, and anchors on the load-carrying capacity of columns. A simplified approach was suggested for the assessment of the load-carrying capacity of the retrofitted columns. A comparison of the predicted load capacity was also made with available models [27,28]. Hosny et al. [19] studied 12 RC columns with rectangular cross-sections of 15 cm × 45 cm and heights of 150 cm. They employed CFRP strips for the confinement of the axially loaded members. They observed that the failure of the FRP-upgraded columns occurred at low FRP strains. The effect of anchorage (by CFRP anchors and steel plates) on the effectiveness of the system was also studied.

Tanwongsval et al. [20] tested FRP-strengthened wall-like columns under concentric load. The column shape was modified by adding a semi-circular section of non-shrink mortar at the two ends of the column section. The columns were then wrapped with GFRP laminates. The performance of this scheme of strengthening was compared with the columns wrapped with GFRP laminates without shape modification. The performance of columns retrofitted after shape modification was reported to be better than for the one strengthened without shape modification. Maalej et al. [21] studied the influence of FRP wrapping on the load-carrying capacity of wall-like concrete columns. The authors extended the load capacity predictions of past studies [29,30] for predicting the load capacity of FRP-strengthened wall-like columns.

Prota et al. [22] studied the effect of FRP strengthening on enhancing the load-carrying capacity of wall-like concrete columns. The authors concluded that the ductility and load capacity could be enhanced by FRP strengthening. At failure, the strains in FRP laminate were much smaller than the fiber fracture strain. De Luca et al. [23] discussed the laboratory testing of three wall-like concrete columns externally confined using GFRP sheets. Two columns were strengthened using a varying number of GFRP sheets. It was concluded that the confinement did not increase the compressive strength of concrete. However, the FRP confinement substantially increased the axial compressive strain at failure and prevented the rebar buckling.

Alsayed et al. [24] studied the response of FRP-strengthened columns with a large cross-sectional aspect ratio under concentric axial loads experimentally. The column section was transformed into an elliptical one using cement mortar; then, it was externally confined by CFRP laminates. The experimental load-compression response was validated with the help of a numerical model. The CFRP confinement increased the load capacity and ductility of the wall-like column. Abbas et al. [25] recently investigated, experimentally and numerically, many other schemes for strengthening wall-like columns. Triantafillou et al. [26] tested 45 wall-like concrete columns with varying section aspect ratios under axial loading. The purpose of the study was to investigate the efficacy of different configurations of wrapping CFRP laminates in combination with shape modification. The authors reported the enhancement in confinement provided by FRP sheets when the number of FRP sheets provided at the section edges was increased.

Concerning the design of strengthening techniques for RC structures using externally bonded FRP composites, design codes, guidelines, or standards have to be accessible by practicing structural engineers. In the literature, ten codes/guidelines were found for designing FRP systems for upgrading RC structures. The ten design documents were developed as follows: two in the United States [31,32], one in Canada [33], one by the European Committee for Standardization [34], one by the European ƒib Task Group 9.3 [35], one in the UK [36], one in Italy [37], two in Asia [38,39], and one by the Egyptian Code Committee [40]. However, three guidelines were found in the literature for strengthening RC structures using near-surface mounted (NSM) techniques. These are ACI 440.2R-17 [31], English TR 55 [36], and Canadian CSA S806-12 [41]. Although the NSM technique is an effective method of retrofitting, the problems of debonding associated with this technique were highlighted by D’Antino and Pisani [42]. In order to address this issue, the authors developed a fracture-based model for the assessment of the bond strength of NSM bars employed for the retrofitting of RC structures. The model was validated using 117 test results available in the literature for different reinforcement types, including rectangular and round bars and strips.

The above-detailed review of the literature illustrates that very limited systematic and detailed experimental studies are available on the monotonic behavior of FRP-confined RC wall-like columns. Previous research has shown that confining rectangular RC columns with FRP jackets can provide marginal increases in the maximum axial compressive load. In fact, design codes and guidelines do not allow the use of FRP confinement for members featuring a side aspect ratio greater than a certain limit (see Ref. [43]). The ACI 440.2R-17 guidelines [31] do not recommend using FRP wrapping for RC columns with a sectional aspect ratio greater than 2.0. The novelty of this study is that large-size wall-like column specimens, strengthened with innovative hybrid NSM/CFRP systems, are tested under concentric loading. This helped in investigating and validating the efficacy of CFRP wrapping in enhancing the behavior of wall-like concrete columns experimentally. In this research, a total of six RC wall-like column specimens of half-scale were cast and tested under concentric axial compression. Two columns were unstrengthened to serve as control specimens, and four were strengthened using different schemes. The 3D numerical models were prepared to predict the response of tested specimens in terms of the load-compression variation and failure modes.

## 2. Experimental Program

The experimental program involves a series of experiments involving strengthening and testing wall-like RC columns in the event of axial load. The key parameter of the investigation was the strengthening scheme. Four schemes for strengthening wall-like RC columns were developed and tested experimentally.

### 2.1. Test Matrix

**Figure 1:**
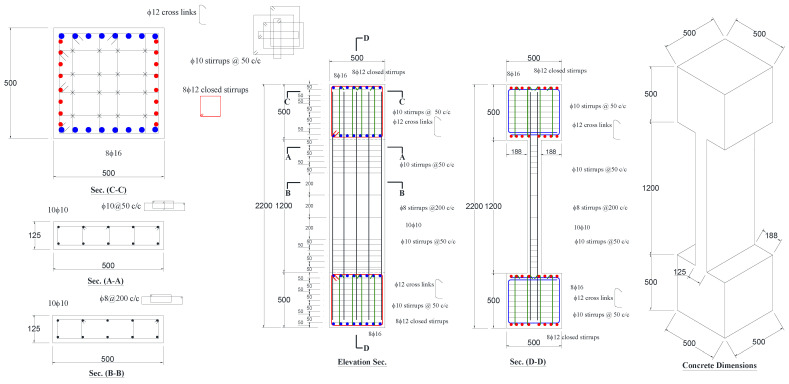
Dimensions and reinforcement detailing of control specimen CW (note: all dimensions are in mm).

A representative prototype wall-like column was selected to be an interior ground-story column of an existing seven-story (G+6) commercial building. Half-scale columns were then selected as test specimens. It should be pointed out that full-scale columns were not selected in this study as the peak load of the full-scale strengthened specimens may exceed the capacity of the available compression testing machine. Moreover, it is clarified from previous research conducted by the authors on FRP-confined concrete [44] that with the same FRP confinement ratio, no significant variations occur in neither compressive strength nor ultimate strain when different sizes of FRP-confined concrete specimens were used. Accordingly, there is no need to introduce a size factor for the test results, which are based on scaled sizes of FRP-wrapped concrete specimens. The test matrix is shown in Table 1. A total of six RC wall-like columns having a cross-sectional aspect ratio of four were cast. Two identical columns (CW) served as control specimens, and the remaining four specimens (SW1, SW2, SW3, and SW4) were upgraded using different schemes. The control specimen (CW) of dimensions 125 × 500 × 1200 mm was designed as shown in Figure 1. The design of the column conforms to the relevant code provisions [45,46]. The columns were reinforced with 10 ф10 longitudinal rebars (1.26% steel), and the transverse ties provided in the middle and the end portions were ф8 at a center-to-center spacing of 200 mm and ф10 at a center-to-center spacing of 50 mm, respectively. The column ends were designed to have bulbs of 500 × 500 × 500 mm size to eliminate the concentration of stresses and to properly distribute the axial load at the ends (Figure 1). The bulbs were reinforced with ф10, ф12, and ф16 steel rebars.

### 2.2. Strengthening Schemes

**Figure 2:**
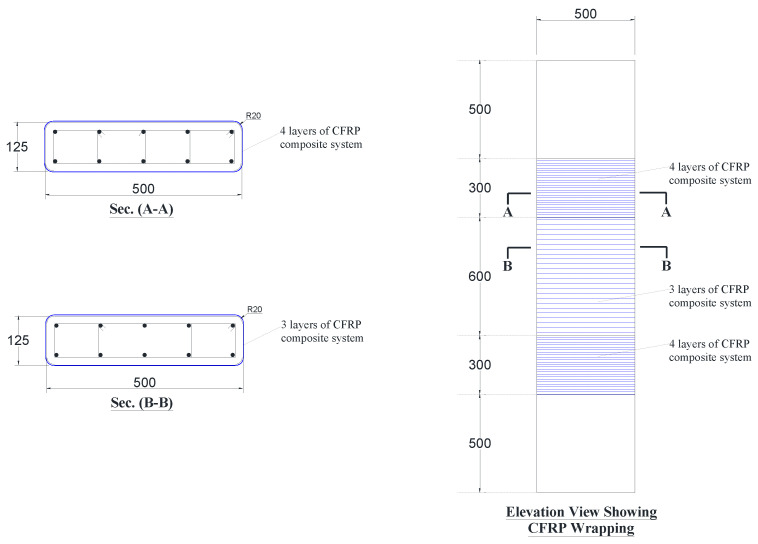
Dimensions and reinforcement detailing of strengthened specimen SW1 (note: all dimensions are in mm).

Four schemes were developed for the strengthening of wall-like columns. The first scheme (scheme-1) employed the conventional externally bonded FRP wrapping of the column with fibers oriented in the circumferential direction. In this scheme, CFRP composite sheets were tried, and the column (SW1) was wrapped with three layers of the sheet in the middle 600 mm length; however, in the end zones of the column, an extra CFRP layer was added to ensure the column failure in the middle portion. The details of this scheme are presented in Figure 2.

**Figure 3:**
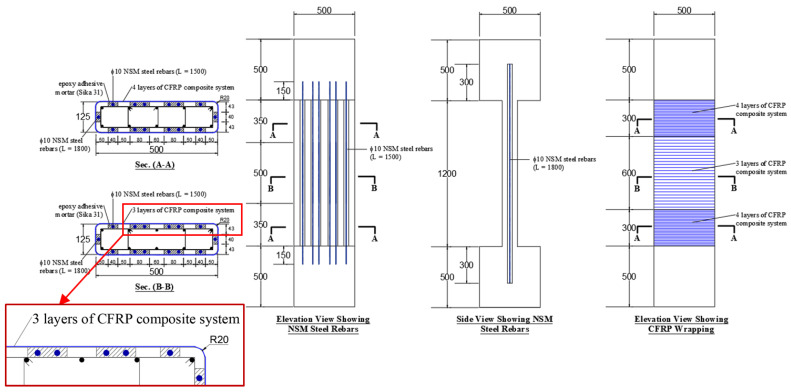
Dimensions and reinforcement detailing of strengthened specimen SW2 (note: all dimensions are in mm).

The second strengthening scheme (scheme-2) employed a combination of vertical NSM steel rebars and externally bonded CFRP wrapping. The provided NSM rebars were 14 ф10, which enhanced the column reinforcement from 1.26% to 3.01%. The provided vertical NSM rebars require transverse ties for preventing their buckling. However, in this scheme of strengthening, CFRP wrapping was utilized to support the NSM rebars laterally. The CFRP wrapping scheme was the same as that adopted for column SW1, i.e., three layers in the central portion and four layers at the two ends. The strengthening details of this scheme are shown in Figure 3. It should be noted that the NSM rebars in this scheme were continuous and connected to the top and bottom RC bulbs with development lengths of 150 mm and 300 mm for the 12 ф10 bars on the long side and the 2 ф10 bars on the short side of the column, respectively (see Figure 3). It is worth mentioning that a shorter development length of 150 mm was selected for the NSM bars on the long side of the column because these bars were developed in compression in the confined concrete of the RC bulbs by drilling and bonding with epoxy adhesive mortar. However, the bars on the short side were developed in compression in the unconfined concrete cover of the end bulbs via preinstalled grooves filled with epoxy adhesive mortar.

**Figure 4:**
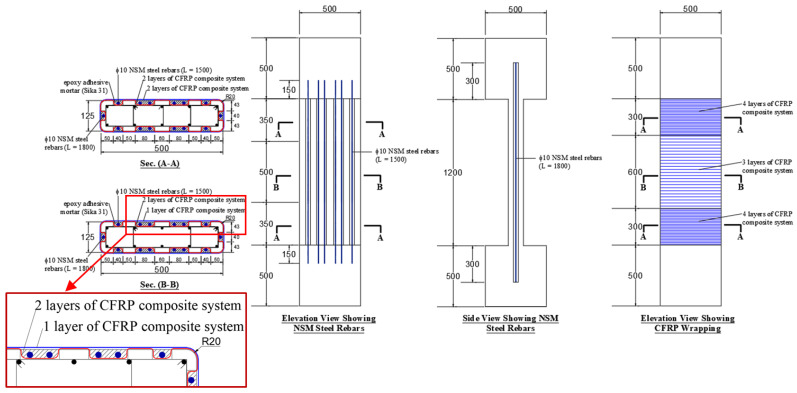
Dimensions and reinforcement detailing of strengthened specimen SW3 (note: all dimensions are in mm).

The third scheme of strengthening wall-like columns is the same as scheme-2 except that the arrangement of the CFRP layers was different. The details of scheme-3 are shown in Figure 4. In this scheme, two of the CFRP layers were bonded to the column surface before the installation of NSM rebars, and these layers were bent inside the NSM grooves, as seen in Figure 4. However, the remaining CFRP layers were bonded to the column surface after the installation of the NSM rebars and filling out the NSM grooves with epoxy adhesive mortar (see Figure 4).

**Figure 5:**
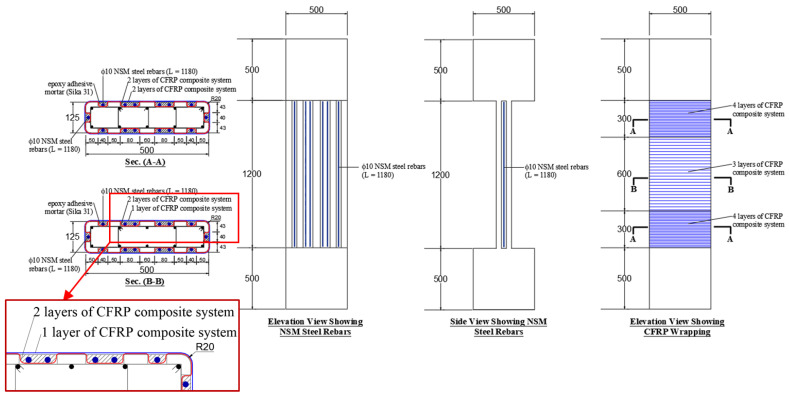
Dimensions and reinforcement detailing of strengthened specimen SW4 (note: all dimensions are in mm).

As seen in Figure 5, scheme-4 is exactly like scheme-3 except that the NSM rebars were disconnected from the top and bottom RC bulbs. The length of the NSM rebars was 20 mm shorter than the column height, and a gap of 10 mm width was provided between the NSM rebars and the RC bulbs, as seen in Figure 5.

### 2.3. Properties of Material

The concrete of the wall-like columns was obtained from a local company with a target compressive strength of 25 MPa. Six standard 150 × 300 mm concrete cylinders were prepared from the mix. Out of the six cylinders, three were tested at 28 days, and the remaining three were tested on the day of testing the columns. Testing of concrete cylinders was as per the ASTM C39/C39M [47]. The average concrete strengths were 26.10 MPa and 29.15 MPa at 28 days and on the day of column testing, respectively.

Locally manufactured deformed rebars of diameters 8 and 10 mm were utilized as steel reinforcement for the column specimens. Steel rebars were tested under uniaxial tension as per the ASTM E8/E8M [48], and the average mechanical properties are listed in Table 2.

Commercially available CFRP composite laminates were utilized for the strengthening of columns. It should be clarified that the properties reported in Table 2 for the CFRP composite system are based on the gross-laminate area and not on the net-fiber area of the system. Three standard tensile coupons were prepared in the lab for the CFRP sheets and then tested as per the ASTM D3039/ D3039M–14 [49]. Table 2 presents the average mechanical properties of the CFRP composite system. It is worth mentioning that the longitudinal tensile strength of CFRP laminates listed in Table 2 is taken as 55% of the tensile strength of flat CFRP test coupons [31]. The 55% reduction is called the FRP strain efficiency factor that accounts for the premature failure of the FRP system, related primarily to stress concentration regions (e.g., corner of wall-like columns) caused by cracking of the concrete as it dilates.

The commercially available structural adhesive mortar (Sikadur-31) was utilized for filling the NSM grooves in specimens SW2, SW3, and SW4. Table 2 presents the mechanical properties of the mortar as provided by the manufacturer’s datasheet.

### 2.4. Preparation of Specimens

**Figure 6:**
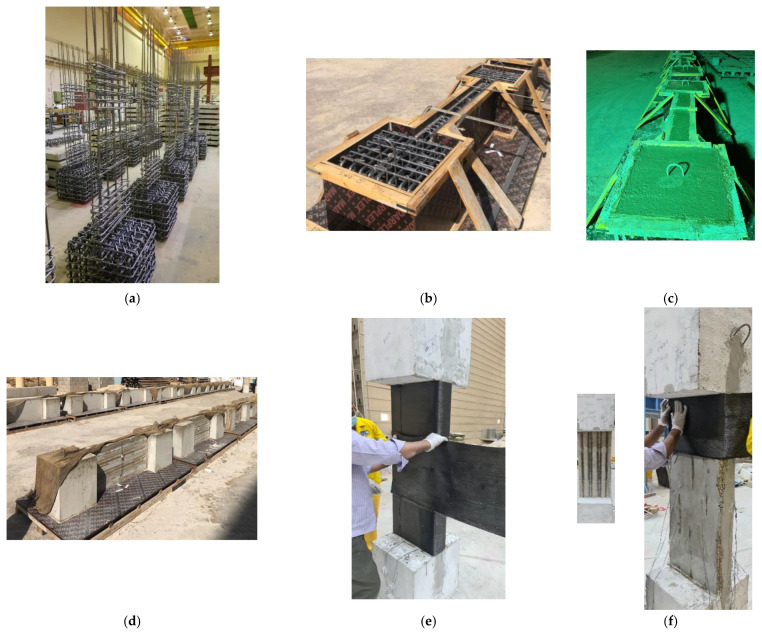
Preparation of test specimens: (**a**) rebar cages; (**b**) reinforcement cages in the formworks before casting of concrete; (**c**) test specimens after casting; (**d**) test specimens ready for testing and strengthening; (**e**) strengthening of column with scheme-1 using CFRP sheets (SW1); and (**f**) strengthening of column with scheme-2 using CFRP sheets combined with connected NSM rebars (SW2).

Figure 6 shows the different steps followed in the preparation of test specimens. The rebar cages of the wall-like columns are illustrated in Figure 6a. Figure 6b,c depict the wall-like columns before and after casting. All specimens were cast simultaneously using ready-mix concrete to avoid variations in concrete batches. Figure 6d shows the test specimens after stripping of forms and curing. The control columns were tested, whereas the columns meant for strengthening were strengthened using the developed schemes, as explained in Section 2.2. It should be noted that in all strengthened specimens the corners were rounded with a radius of 20 mm to reduce the concentration of CFRP stresses at corners (see Figure 2, Figure 3, Figure 4 and Figure 5).

In strengthening scheme-1, sandpaper was employed to grind the column surface. Subsequently, sandblasting was performed to take away all the unevenness on the surface, which was afterward cleaned with the help of acetone. The wet layup procedure was adopted in wrapping CFRP sheets. Figure 6e shows the process of strengthening the column with scheme-1 using CFRP sheets (specimen SW1).

In other strengthening schemes (specimens SW2, SW3, and SW4), NSM rebars were used, and they were provided in longitudinal grooves in the concrete cover. These grooves were made by attaching strips of polystyrene foams to the column during casting (Figure 6d). In scheme-2, the reinforcing rebars were planted into these grooves and bonded with an epoxy adhesive mortar (Sikadur 31), as presented in Figure 6f. After the curing of adhesive mortar, CFRP sheets were wrapped around the column, as seen in Figure 6f. Nevertheless, for schemes 3 and 4, two CFRP layers were bonded to the column surface before the installation of NSM rebars, and these layers were bent inside the NSM grooves. Thereafter, the NSM rebars were inserted into the grooves and bonded with epoxy mortar (Sikadur-31). After curing of the epoxy mortar, the surface of the mortar was sandblasted and then cleaned using acetone liquid. Subsequently, the remaining CFRP layers were bonded to the column’s surface. It should be noted that in schemes 2 and 3, the 12 ф10 NSM rebars on the long side were connected to the top and bottom bases by drilling 150 mm deep holes and then bonding the rebars using epoxy mortar (Sikadur-31). However, the 2 ф10 NSM rebars on the short side were connected to the top and bottom bases by bonding them in the preinstalled concrete cover grooves (with a development length = 300 mm) using epoxy adhesive mortar. In scheme-4, the NSM rebars were disconnected from the top and bottom bases.

### 2.5. Testing Setup and Instrumentation Layout

**Figure 7:**
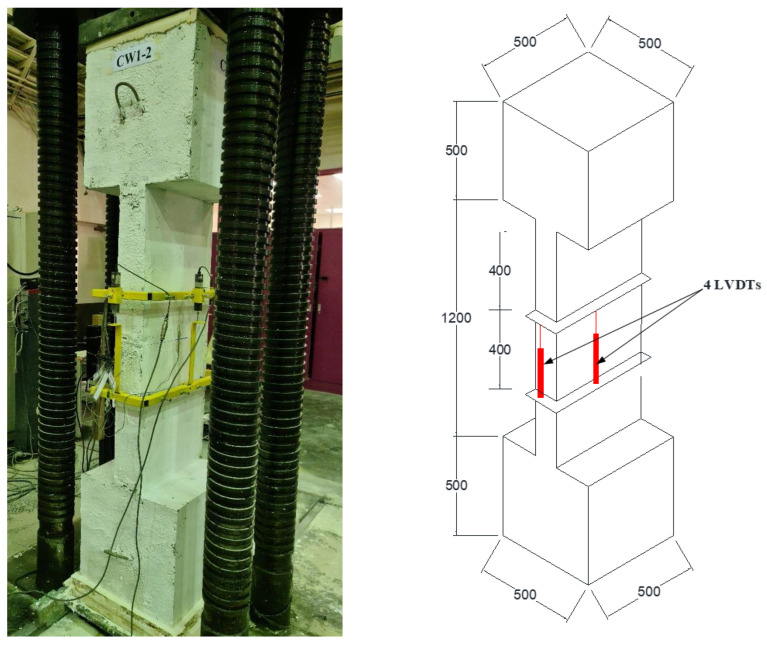
Test setup with instrumented specimen ready for testing.

**Figure 8:**
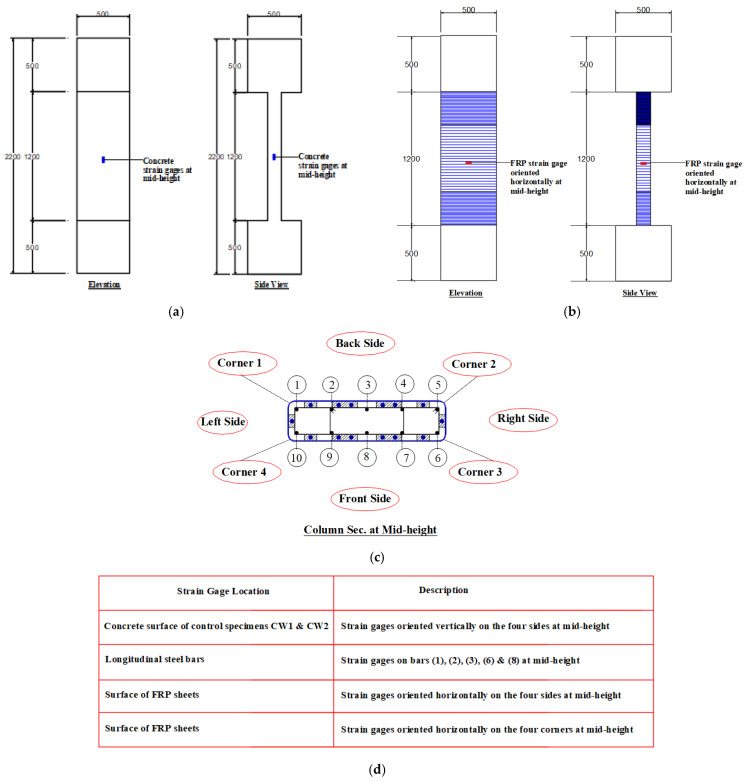
Layout of strain gages for test specimens (note: all dimensions are in mm): (**a**) locations on concrete surface for control specimens CW1 and CW2; (**b**) locations on FRP surface for strengthened specimens; (**c**) numbering of steel rebars for all specimens; and (**d**) table showing locations for different parts of test specimens.

Figure 7 presents the instrumentation layout and testing setup for column specimens. The concentric axial compressive load was applied to the columns via a compression testing machine at a rate of 0.5 mm/min. For measuring the axial displacement of the specimen, four LVDTs (linear variable displacement transducers) were attached to the central part of the column, as illustrated in Figure 7. Strain gages were also used to measure strains in different parts of the specimen (concrete, steel rebars, and CFRP sheets). Figure 8 shows the location of strain gauges installed on different parts of the test specimens. The test results were monitored using a data acquisition system.

## 3. Discussion of Experimental Results

**Figure 9:**
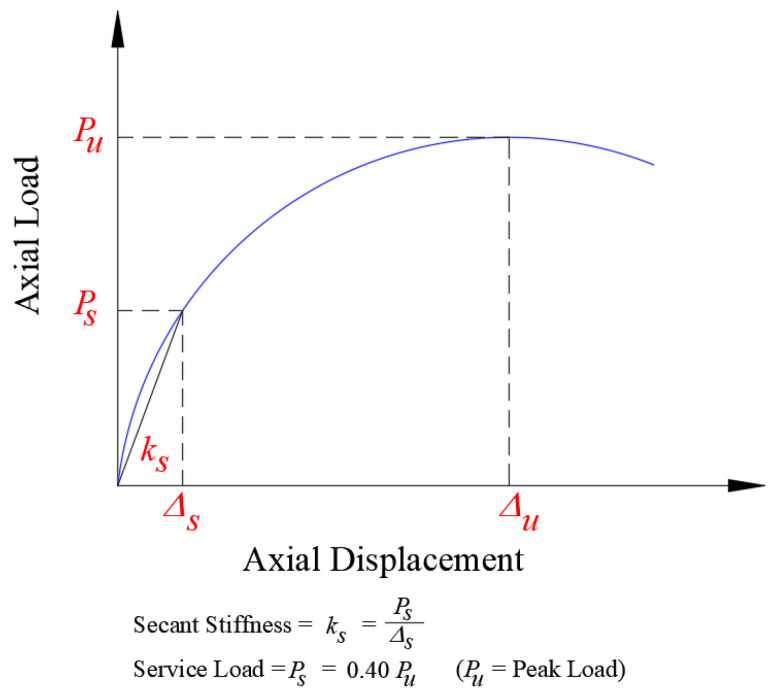
Calculation of secant stiffness for tested columns [7,50,51].

**Figure 10:**
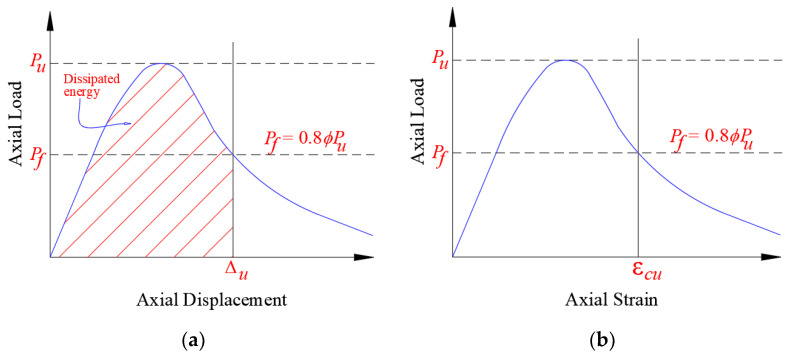
Definition of ultimate state for test columns: (**a**) dissipated energy; (**b**) ultimate axial concrete strain.

Table 3 depicts the key test results of the load-displacement response for test columns with regard to load and axial displacement at the onset of main rebar yielding, peak load, axial displacement at service load level, axial displacement at maximum load, axial displacement at the ultimate state level, stiffness at service load level, and dissipated energy. In the current research, the column stiffness (*K_s_*) was computed as the ratio of service axial load (assumed as 40% of the maximum load [7,50,51]) to the related axial displacement (see Figure 9). As shown in Figure 10a, the energy dissipated (*E_u_*) was assessed as the area under the load-displacement envelope up to ultimate displacement. The ultimate state was taken as that corresponding to concrete crushing, as presented in Figure 10b, and it is assumed as that corresponding to a failure load of $P_{f}=0.8\;\phi P_{u}$ where *ϕ* is the resistance factor for sections under concentric axial compression (= 0.65 as per both ACI 318-19 code [45] and SBC 304-18 code [46]). The reduction factor of 0.8 was taken as per the requirements of both ACI 318-19 and SBC 304-18 codes to account for the minimum eccentricity for tied columns. In this regard, it is assumed in this study that the column reaches its ultimate limit state if its load-carrying capacity is less than the design ultimate capacity calculated by the code.

Table 4 displays the key experimental results of the axial stress-strain response for test specimens with respect to: peak average concrete strength ($f_{c-avg}^{’}$), maximum actual concrete strength ($f_{c-act}^{’}$), axial concrete strain at peak stress ($\varepsilon_{c-pu}$), ultimate axial concrete strain ($\varepsilon_{cu}$), strains in main and NSM steel rebars, respectively, at peak load ($\varepsilon_{s-pu}\&\varepsilon_{s,NSM-pu}$), and peak horizontal strain in CFRP laminates of strengthened specimens ($\varepsilon_{FRP,u}$). The peak average concrete strength was calculated from

(1) $$f_{c-avg}^{’}=\frac{P_{u}}{A_{g}}$$

However, the peak actual concrete strength is computed from

(2) $$f_{c-act}^{’}=\frac{P_{u}-A_{st}f_{y-st}-A_{NSM}f_{y-NSM}}{A_{g}-A_{st}-A_{NSM}}$$

where *P_u_* is the maximum load; *A_g_* and *A_st_* are, respectively, the area of column section and main steel rebars; *f_y-st_* and *f_y-NSM_* are, respectively, the yield strength of longitudinal and NSM rebars; and *A_NSM_* is the area of NSM rebars.

**Figure 11:**
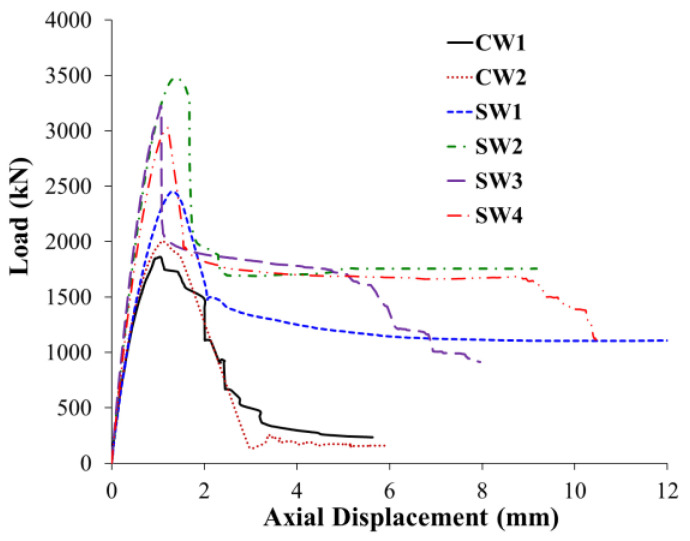
Experimental load versus axial displacement plots for tested columns.

**Figure 12:**
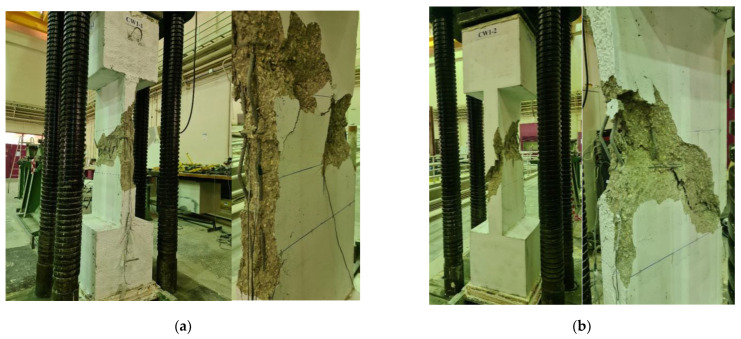
Failure mode of control columns: (**a**) specimen CW1; (**b**) specimen CW2.

**Figure 13:**
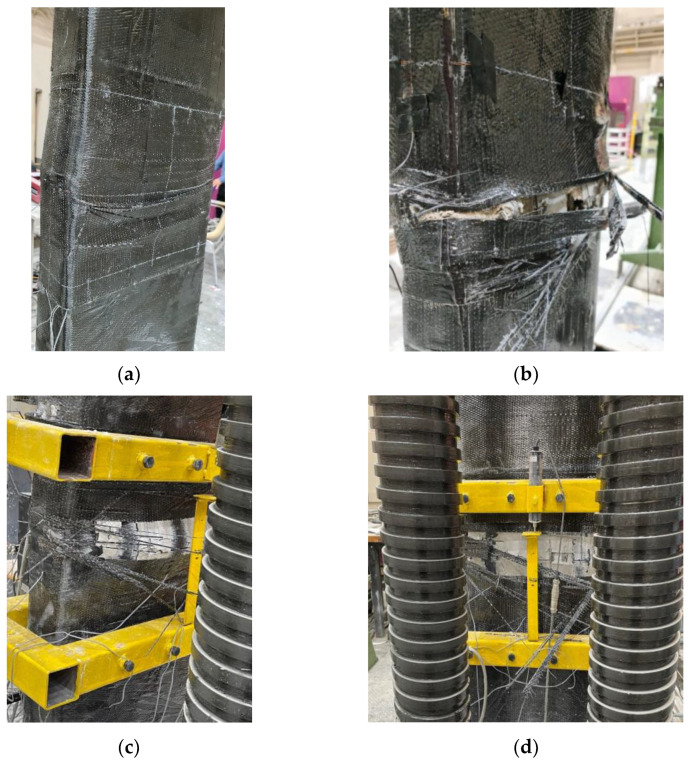
Failure mode of strengthened columns: (**a**) SW1; (**b**) SW2; (**c**) SW3; (**d**) SW4.

Figure 11 shows the load-displacement plots of the tested specimens. Figure 12 and Figure 13 present the modes of failure for unstrengthened and strengthened columns, respectively. Following is a discussion of the experimental results for control and strengthened RC wall-like column specimens.

### 3.1. Unstrengthened Columns

As illustrated in Figure 11 and Table 3, the maximum load achieved for the two control (i.e., unstrengthened) columns CW1 and CW2 was 1862 and 2006 kN, respectively, with an average load of 1934 kN. After reaching the peak, there was a continuous decrease in the axial capacity until it dropped to almost zero. Comparing the load-displacement plots for the two columns shown in Figure 11 indicates that the test results are consistent. Figure 12 presents the failure mode of the unstrengthened columns CW1 and CW2. The failure of the two columns showed typical brittle failure, which was initiated by concrete cover spalling when the axial compressive strain exceeded the crushing strain of concrete. This led to the buckling of longitudinal column rebars and consequent total failure owing to concrete crushing in the central portion of the column height (Figure 12).

### 3.2. Strengthened Columns

#### 3.2.1. Column Strengthened with Scheme-1 (SW1)

As mentioned previously, specimen SW1 was strengthened by the external wrapping of three layers of CFRP sheets within the middle 600 mm portion of the column. As depicted in Figure 11 and Table 3, the maximum load attained for this column was 2451 kN, which is 27% higher than the average peak load of unstrengthened columns. As the axial load increased, the concrete expanded laterally, owing to Poisson’s effect. At the peak load of 2451 kN, excessive concrete dilation occurred so that the CFRP sheets could not control the concrete expansion due to the large depth-to-width ratio of the column section. Accordingly, bulging of the CFRP sheets was noticed in the upper part of the middle 600 mm portion of the column. Subsequently, there was a rapid decrease in the axial capacity of the column, but it stopped at about 1400 kN and remained almost flat (see Figure 11). The bulging of the column section resulted in the buckling of longitudinal column rebars, which caused the rupture of CFRP sheets at a corner of the column section (Figure 13a).

#### 3.2.2. Column Strengthened with Scheme-2 (SW2)

As outlined previously, this column was upgraded by 14 ф10 mm continuous NSM steel rebars in combination with the wrapping of three CFRP layers around the middle portion of the column. As seen from Figure 11 and Table 3, the enhancement in maximum load for this scheme was about 80% compared to the average peak load of control columns. The maximum load achieved for the strengthened column was 3478 kN, which is 42% higher than the load-carrying capacity of the column strengthened using the CFRP wrapping scheme (i.e., SW1). Thus, this strengthening scheme is quite efficient at enhancing the axial compression response of RC wall-like columns.

Figure 13b shows the mode of failure of the wall-like column SW2. As the axial load increased, the concrete expanded laterally, owing to Poisson’s effect. At the peak load of 3478 kN, excessive concrete dilation occurred so that the CFRP sheets and the NSM rebars could not control the concrete expansion due to the large aspect ratio of the column section. Accordingly, buckling of NSM rebars and bulging of the CFRP sheets were noticed in the upper part of the middle 600 mm portion of the column. Owing to NSM rebar buckling, there was a rapid drop in the axial capacity of the column to about 2000 kN (close to the average peak load of control specimens), but it stopped at about 1700 kN and remained almost flat (see Figure 11). Increasing the axial displacement of the specimen caused concrete crushing and buckling of main steel rebars, which led to the rupture of CFRP laminates on the smaller side of the column section (see Figure 13b).

#### 3.2.3. Column Strengthened with Scheme-3 (SW3)

As detailed before, this strengthening scheme is the same as scheme-2 except that two of the three CFRP wrapping layers were bonded to the inside surface of the NSM grooves before the installation of the NSM rebars, and the remaining CFRP layer was later bonded to the outer column surface. As seen from Figure 11 and Table 3, the enhancement in maximum load for this scheme was about 67% compared to the average peak load of control columns. The peak load achieved for the strengthened column SW3 was 3225 kN, which is 32% higher than the maximum load of upgraded specimen SW1; however, it was less than the peak load of upgraded specimen SW2 by about 7%. Thus, this strengthening scheme is more effective than the wrapping scheme-1; yet it is slightly less effective than scheme-2. The reduced efficiency of this scheme compared to scheme-2 is attributed to the less confinement provided to the column section by CFRP wrapping. The area of the NSM grooves (about 18% of the column section) was only confined by a single layer of CFRP laminates, and the remaining 82% of the section was confined by three CFRP layers.

Figure 13c presents the failure mode of strengthened column SW3. As the axial load increased, the concrete expanded laterally, owing to Poisson’s effect. At the peak load of 3225 kN, excessive concrete dilation occurred so that the CFRP sheets and the NSM rebars could not control the concrete expansion due to the large aspect ratio of the column section. Consequently, buckling of NSM rebars and bulging of the CFRP sheets were noticed in the middle 600 mm portion of the column. This caused a sudden drop in the axial capacity of the column to about 2000 kN (close to the average peak load of control specimens) but stopped at about 1700 kN and remained almost flat up to an axial displacement of about 5 mm (see Figure 11). Increasing the axial displacement of the column caused the crushing of both concrete and epoxy adhesive mortar and buckling of main rebars, which led to the rupture of CFRP laminates that started on the smaller side of the column section and subsequently spread out to the longer side as illustrated in Figure 13c.

#### 3.2.4. Column Strengthened with Scheme-4 (SW4)

As noted previously, this strengthening scheme is the same as scheme-4 except that the 14 ф10 NSM steel rebars were disconnected from the top and bottom bases. As seen from Figure 11 and Table 3, the enhancement in maximum load for scheme-4 was about 57% compared to the average peak load of control columns. The maximum load achieved for the strengthened column SW4 was 3027 kN, which is 24% higher than the axial capacity of strengthened column SW1; however, it is less than the peak load of upgraded specimens SW2 and SW3 by about 13% and 6%, respectively. The reduced efficiency of this scheme compared to scheme-2 is attributed to the less confinement provided to the column section by CFRP wrapping. However, compared to SW3, the 6% reduction in the peak load of specimen SW4 could be owing to the reduced compressive strength of the epoxy mortar used in the NSM grooves.

Figure 13d presents the failure mode of strengthened specimen SW4. It is identified that the failure modes of specimens SW3 and SW4 are almost identical. As the axial load increased, the concrete expanded laterally, owing to Poisson’s effect. At the peak load of 3027 kN, excessive concrete dilation occurred so that the CFRP sheets and the NSM rebars could not control the concrete expansion due to the large aspect ratio of the column section. Accordingly, buckling of NSM rebars and bulging of the CFRP sheets were noticed in the middle 600 mm portion of the column. This caused a sudden drop in the axial capacity of the column to about 1900 kN (close to the average peak load of control specimens) but stopped at about 1700 kN and remained almost flat up to an axial displacement of about 9 mm (see Figure 11). The further increase in the compression of the column caused the crushing of concrete and epoxy adhesive mortar in the NSM grooves and buckling of the main longitudinal steel rebars of the column, which led to the rupture of CFRP sheets in the middle 600 mm portion of the column, as shown in Figure 13d.

### 3.3. Comparison of Strengthening Schemes

**Figure 14:**
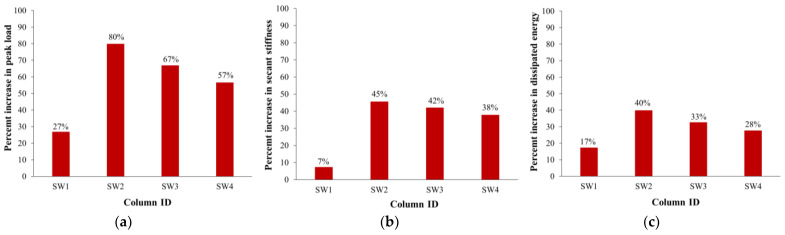
Comparison of strengthened columns with respect to percent enhancement in: (**a**) maximum load; (**b**) stiffness; and (**c**) energy dissipated.

The effect of the strengthening scheme on the enhancement in maximum load, stiffness, and energy dissipated is illustrated in Figure 14. It is identified that the external wrapping of three CFRP layers in scheme-1 was not effective at enhancing the axial capacity of wall-like specimens, as the increase was only moderate by about 27%. However, using vertical NSM rebars in combination with the wrapping of three CFRP layers (schemes 2, 3, and 4) was very efficient at enhancing the axial capacity of the wall like-columns by about 57% to 80%, as seen in Figure 14a. The largest ultimate load increase of 80% was provided by scheme-2, in which the three CFRP layers were attached to the outer column surface, and the vertical NSM rebars were anchored to the top and bottom RC boxes, as detailed earlier. For scheme-3, the peak load increase was 13% less than scheme-2. This is because the wrapping scheme used in scheme-3 (bonding two CFRP layers inside the NSM rebars and one layer on the outer column surface) was less efficient and gave less confinement than that used in scheme-2 (wrapping three CFRP layers on the outer column surface). It was also clarified that even though scheme-4 is the same as scheme-3 except that the NSM rebars were disconnected from the top and bottom RC bulbs, the peak load increase of scheme-4 was 10% less than scheme-3. As noted from the test observations, the disconnected NSM rebars in specimen SW4 were fully activated during the loading process and continued to share in carrying the axial load until their buckling, as discussed earlier. Therefore, the 10% reduction in the peak load of scheme-4 compared to scheme-3 could be owing to the low strength of the adhesive mortar in specimen SW4 compared with specimens SW2 and SW3.

As seen from Figure 14b, the external wrapping of three CFRP layers in scheme-1 had a minor effect on enhancing the secant stiffness of the wall-like column as the increase was limited to 7%. However, using vertical NSM rebars in combination with the wrapping of three CFRP layers (schemes 2, 3, and 4) was very efficient at enhancing the secant stiffness of the wall like-columns by about 38% to 45%, as seen in Figure 14b, with the highest increase of 45% provided by scheme-2. It should be mentioned that it is favorable to increase the axial stiffness of upgraded wall-like columns at service loads because it will decrease the creep deformation and hence axial shortening in RC buildings.

As detailed before, the energy dissipated at the ultimate state was computed for each specimen, and its percent increase owing to strengthening was evaluated, as seen in the bar chart of Figure 14c. It is identified that for scheme-1 with the external wrapping of three CFRP layers, the increase in the dissipated energy was limited to 17%. Nevertheless, for schemes 2, 3, and 4, in which vertical NSM rebars were combined with CFRP wrapping, the enhancement in the energy dissipated varied from 28% to 40%, with the highest increase provided by scheme-2 (see Figure 14c).

## 4. Finite Element Analysis

In addition to the experimental program, 3D nonlinear finite element analysis (FEA) was carried out to predict the response of concentrically loaded unstrengthened and strengthened RC wall-like columns. For this purpose, the commercial software LS-DYNA [52] was utilized.

### 4.1. Model Geometry and Mesh Generation

**Figure 15:**
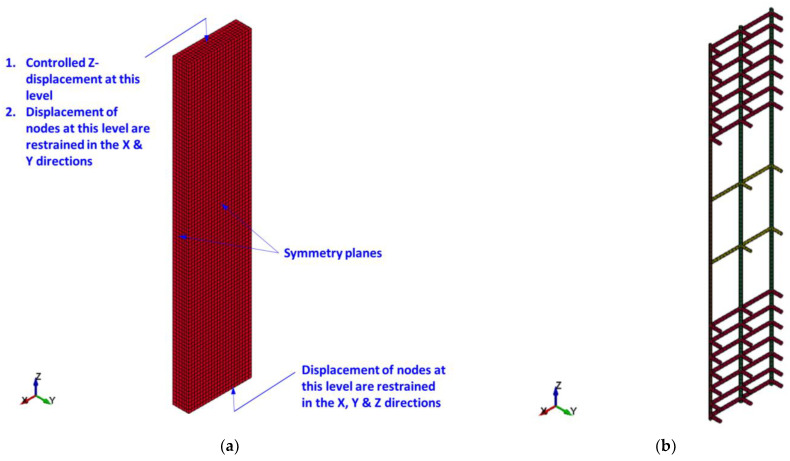
FE mesh for control specimen CW: (**a**) concrete volume; (**b**) reinforcement cage.

**Figure 16:**
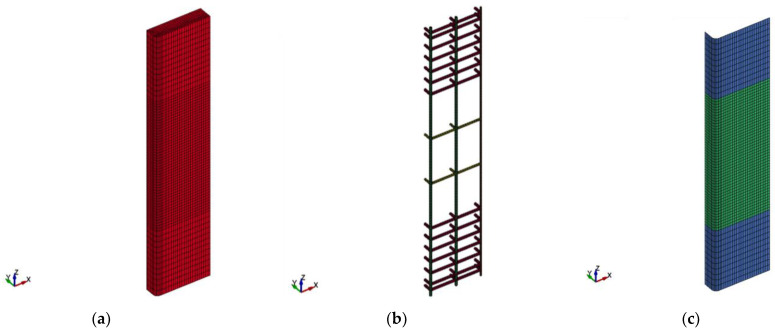
FE mesh for strengthened specimen SW1: (**a**) concrete volume; (**b**) reinforcement cage; and (**c**) CFRP sheets.

**Figure 17:**
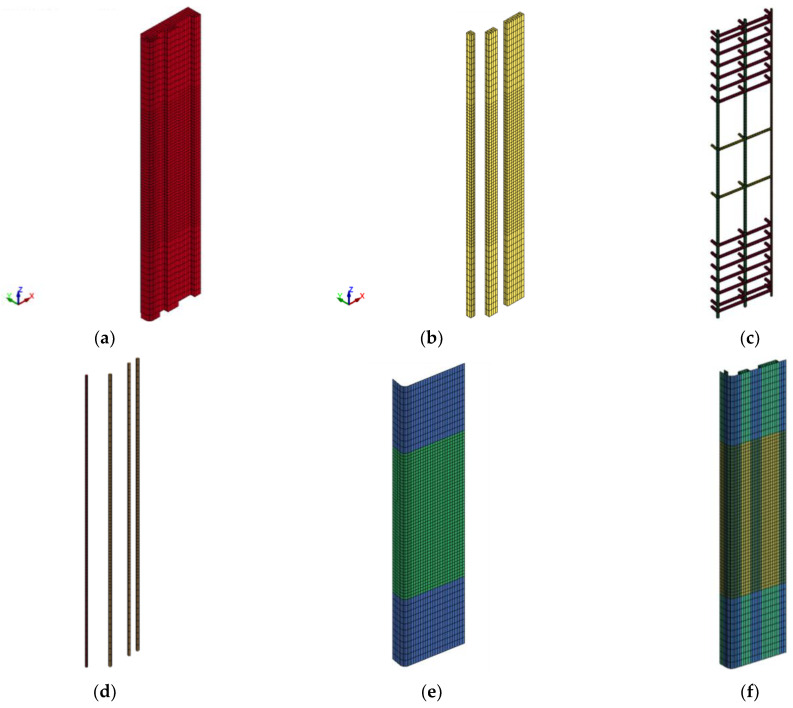
FE mesh for strengthened columns SW2, SW3, and SW4: (**a**) concrete volume; (**b**) epoxy adhesive mortar; (**c**) reinforcement cage; (**d**) NSM rebars; (**e**) CFRP sheets for specimen SW2; and (**f**) CFRP sheets for specimens SW3 and SW4.

Figure 15, Figure 16 and Figure 17 present the FE mesh for RC wall-like column specimens. It should be noted that one-quarter of the column was numerically simulated, considering its symmetry about both XZ and YZ planes. It should also be noted that the top and bottom boxes at the ends of the specimen were not included in the FE model geometry. This is because these boxes were heavily reinforced, and their behavior was linear elastic throughout the testing with no concrete cracking and/or crushing. This was also done to reduce the number of elements and therefore save the solution time. For capturing the actual performance of test specimens, 3D FE models were developed for the columns. In this regard, one-point integration solid elements of eight nodes were utilized for epoxy mortar and concrete. For transverse and main rebars, two-node beam elements were employed. FRP sheets were represented by four-node shell elements of the Belytschko-Tsay algorithm [53]. As slippage at rebars-to-concrete interaction was not noticed during the testing of wall-like columns, the perfect bond was modeled at the interface of concrete with transverse and main steel rebars. Since FRP wrapping of RC columns is considered a contact-critical application, the full bond behavior was simulated at the concrete-to-FRP interaction. Additionally, in strengthened specimens SW2, SW3, and SW4, slippage was ignored at the interaction of concrete with epoxy adhesive mortar. For control column CW, the mesh size ranges from 9.3 mm to 12.5 mm; however, for strengthened columns, the mesh size varies from 2.3 to 25 mm. It has been found that more reduction in the size of elements beyond that illustrated in Figure 15, Figure 16 and Figure 17 has a minor influence on the outputs; nevertheless, it may greatly increase the solution time, which is unfavorable.

### 4.2. Material Modeling

The concrete damage model (type 72R3) was utilized to represent both epoxy mortar and concrete materials. This model was developed in Refs. [54,55,56]. In this constitutive model, three individual failure surfaces are employed to define the deviatoric strength of concrete material. The different model parameters can be automatically calculated by the software from the uniaxial compressive concrete strength. For simulating steel rebars, the plasticity model type 24 was employed, and the stress versus strain behavior in this model was simulated by a bilinear curve. The FRP composite laminates were represented using the enhanced composite damage model (type 54–55). The failure criterion of Chang and Chang [57] was used along with this model. Table 2 shows the material properties used in the FE modeling.

### 4.3. Loading Protocol and Boundary Conditions

**Figure 18:**
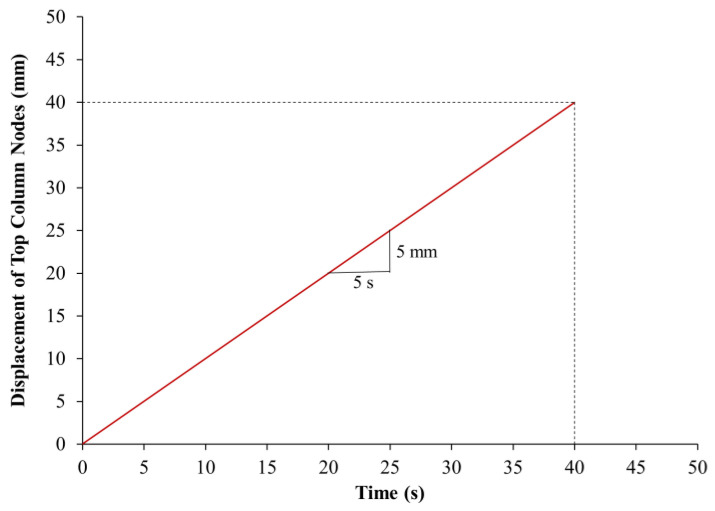
Prescribed axial displacement versus time curve used in the FE modeling.

The extreme bottom nodes of each column were prevented from displacement in the X, Y, and Z directions to simulate the test boundary conditions. However, for the extreme top nodes, the displacements in the X and Y directions only were prevented in order to allow for the vertical Z-displacement. As presented in Figure 15, boundary conditions simulating symmetry were assigned for the two symmetry planes of the one-quarter model. A prescribed, Z-displacement versus time history curve was assigned for the extreme top nodes of the model to simulate the displacement-controlled loading employed in the tests, as illustrated in Figure 18.

## 5. Discussion of FEA Results

**Figure 19:**
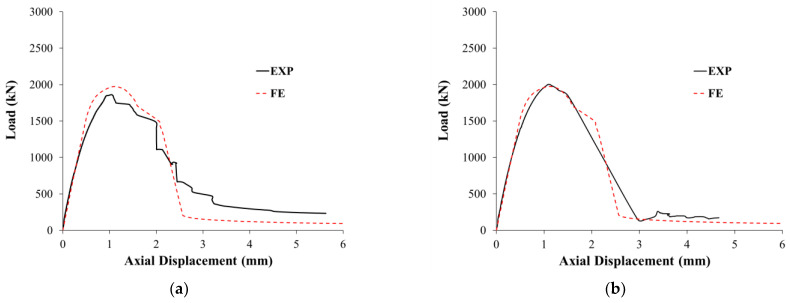
Experimental versus FE load-displacement curves for: (**a**) control specimen CW1; (**b**) control specimen CW2.

**Figure 20:**
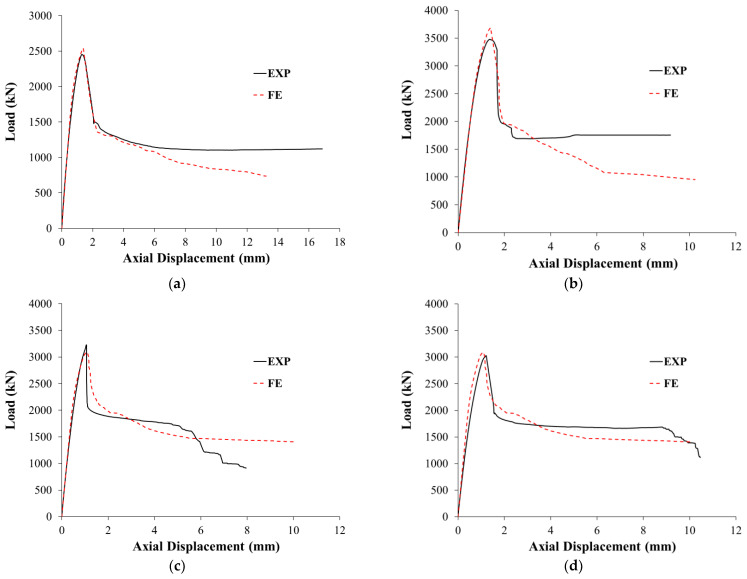
Experimental versus FE load-displacement curves for strengthened specimens: (**a**) SW1; (**b**) SW2; (**c**) SW3; (**d**) SW4.

Comparisons between experimental and FEA results are summarized in Table 3 for test columns with regard to load and axial displacement at the yielding of main rebars, peak load, axial displacement at service load level, axial displacements at maximum load and ultimate state, stiffness at service load level, and dissipated energy. As clarified from the table, the errors in the prediction of yield and peak loads are 2% to 8% and 2% to 6%, respectively. For displacement at service load, displacement at yield load, displacement at maximum load, and displacement at ultimate state, the prediction errors varied from 1% to 10%, 1% to 14%, 2% to 10%, and 1% to 12%, respectively. Nevertheless, the prediction errors of stiffness at service load level and dissipated energy are 0% to 5% and 0% to 11%, respectively. Comparisons of experimental and FE load versus displacement plots for unstrengthened and strengthened columns are presented in Figure 19 and Figure 20, respectively.

Table 4 displays comparisons between the experimental and numerical stress-strain results for test columns with respect to peak average concrete strength ($f_{c-avg}^{’}$), maximum actual concrete strength ($f_{c-act}^{’}$), axial concrete strain at peak stress ($\varepsilon_{c-pu}$), ultimate axial concrete strain ($\varepsilon_{cu}$), axial strains in main and NSM steel rebars at peak load $\left(\varepsilon_{s-pu}\;\&\;\varepsilon_{s,NSM-pu}\right)$, and peak horizontal strain in CFRP laminates of strengthened specimens ($\varepsilon_{FRP,u}$). As illustrated from the table, the errors in the numerical assessment of peak average and actual concrete strengths ranged from 1% to 6% and 2% to 8%, respectively. For concrete strain at ultimate stress and ultimate concrete strain, the prediction errors varied from 2% to 10% and 1% to 12%, respectively. The strain in the longitudinal steel rebars at maximum load was numerically predicted with an error ranging from 0% to 19%. As seen from Table 4, for strengthened specimens, the peak horizontal strains in CFRP laminates were numerically predicted with errors varying from 1% to 19%.

Following is a detailed discussion of the experimental versus FEA results for control and strengthened specimens.

### 5.1. Unstrengthened Columns

**Figure 21:**
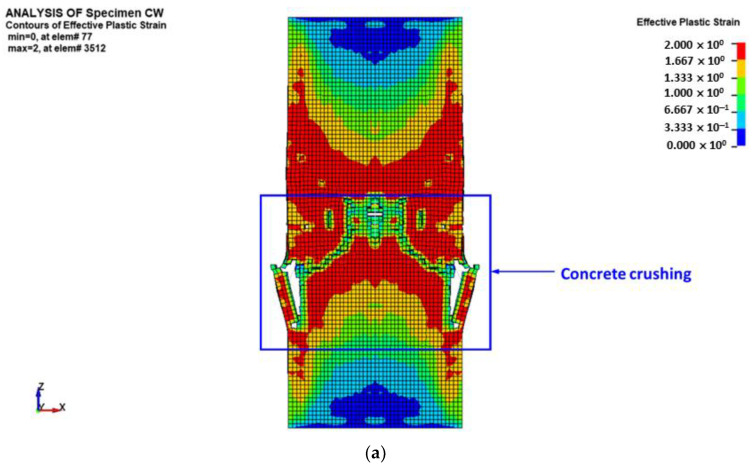

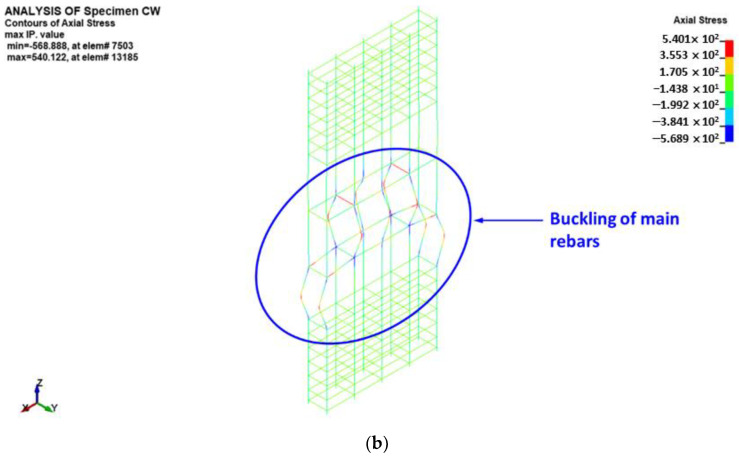
FE mode of failure for control specimen CW: (**a**) effective plastic stress contours for concrete elements; (**b**) axial stress contours for rebar elements.

The predicted failure mode of unstrengthened column CW is presented in Figure 21. The predicted failure modes are presented in terms of damage contours for concrete elements and axial stress contours for beam elements of reinforcement cage (longitudinal and transverse steel rebars). For concrete elements, the damage contours are represented by the effective plastic strain contours, and they range from 0 (shown in blue for the case of no damage) to 2 (shown in red for the case of full damage). As seen in Figure 21, the numerically predicted modes of failure matched well the test results detailed previously for columns CW1 and CW2. It is identified that failure occurred due to concrete crushing (Figure 21a) and buckling of longitudinal rebars (Figure 21b) in the middle 600 mm portion of the column.

Experimental versus FE load-displacement curves for control columns CW1 and CW2 are illustrated in Figure 19a,b, respectively. As noted, a good match was obtained between the experimental and predicted load-displacement curves for unstrengthened specimens CW1 and CW2. The softening behavior was successfully modeled, which proves the precision of the constitutive models used for concrete and steel rebars.

### 5.2. Strengthened Column SW1

**Figure 22:**
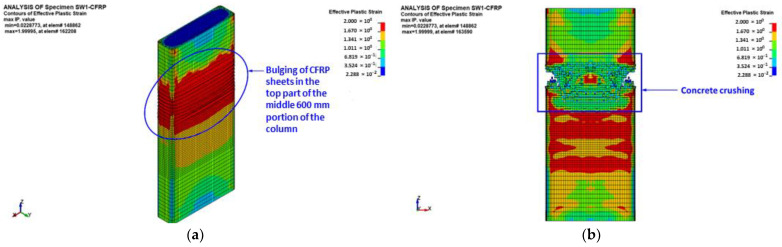

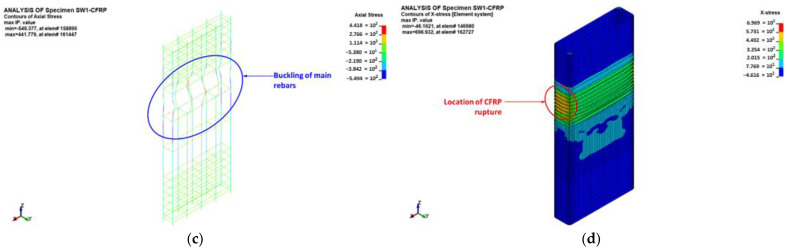
FE mode of failure for strengthened specimen SW1: (**a**) bulging of CFRP sheets at peak load; (**b**) effective plastic stress contours for concrete elements; (**c**) axial stress contours for rebar elements; and (**d**) X-stress contours for shell elements of CFRP sheets.

Figure 22 presents FE failure modes for strengthened column SW1, in which the middle 600 mm portion was upgraded by three CFRP layers. The predicted failure modes are presented in terms of X-stress contours (in the local coordinate system) for CFRP shell elements, damage contours for concrete elements, and axial stress contours for beam elements of the reinforcement cage. As seen in Figure 22, the predicted failure modes matched well the test observations detailed previously in Section 3. Column failure started with bulging of the CFRP laminates in the top part of the central 600 mm length of the specimen. The bulging was simultaneous with a sudden drop in the axial capacity of the specimen. The ultimate failure mode was owing to concrete crushing followed by the buckling of main steel rebars. With increased axial displacement, rupture of CFRP laminates occurred near the corner of the column section, as illustrated in Figure 22.

Figure 20a shows a comparison between experimental and predicted load versus displacement plots for column SW1. It is clear that the two curves agreed well with each other. The softening behavior was successfully modeled, which proves the precision of the used constitutive models for concrete, steel rebars, and CFRP sheets. As seen from Table 3, this strengthening scheme was predicted to have a limited enhancement in the axial capacity, as the maximum predicted load of specimen SW1 was only 29% more than that of control specimen CW.

### 5.3. Strengthened Column SW2

**Figure 23:**
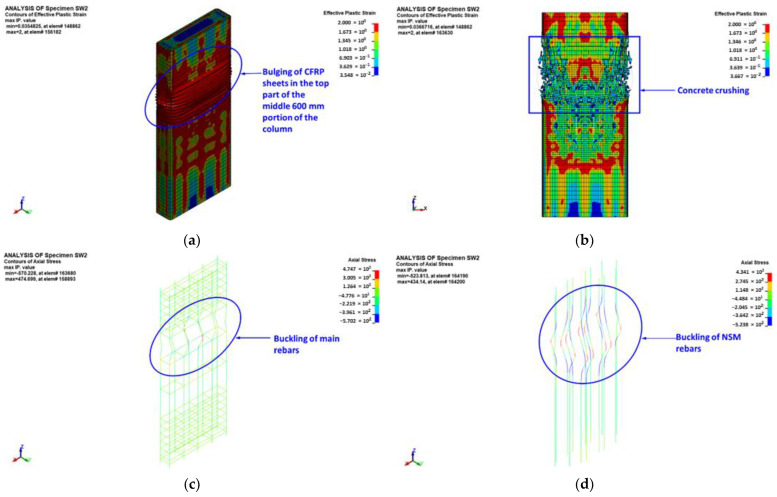

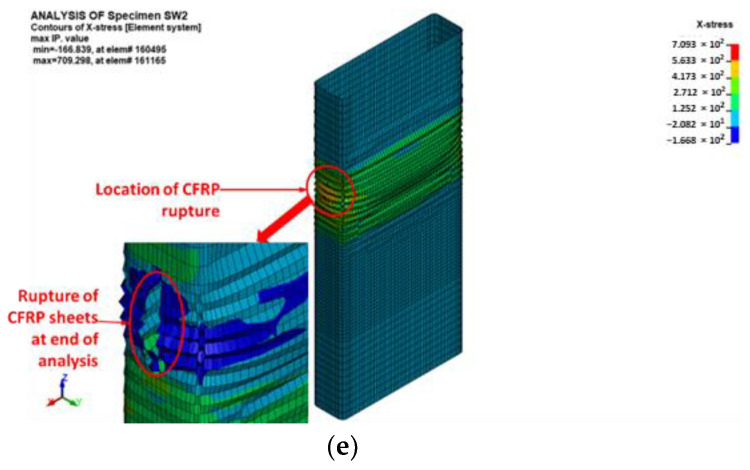
FE mode of failure for strengthened specimen SW2: (**a**) bulging of CFRP sheets at peak load; (**b**) effective plastic stress contours for concrete elements; (**c**) axial stress contours for main rebar elements; (**d**) axial stress contours for NSM rebar elements; and (**e**) X-stress contours for shell elements of CFRP sheets.

Figure 23 shows the numerically predicted failure modes for strengthened column SW2, in which the middle 600 mm portion was strengthened by a combination of three CFRP layers and 14 ф10 continuous NSM rebars. The predicted failure modes are presented in terms of X-stress contours (in the local coordinate system) for CFRP shell elements, effective plastic strain contours for concrete elements, and axial stress contours for beam elements of the reinforcement cage. As seen in Figure 23, the predicted failure modes matched well the test observations detailed in Section 3. The failure of specimen SW2 started by bulging of the CFRP layers in the top part of the middle 600 mm portion of the column. The bulging was concurrent with a rapid decrease in the axial capacity of the column. The ultimate failure was located in the top part of the middle 600 mm portion of the specimen, and it was owing to concrete crushing followed by a buckling of main and NSM steel rebars. With increased axial displacement, rupture of CFRP laminates occurred in the short side of the column section, as seen in Figure 23. This was evidenced by the predicted peak horizontal strain in the CFRP sheets listed in Table 4, as it was almost the same as the rupture strain of the CFRP composite system.

Experimental versus FE load-displacement curves for strengthened column SW2 are shown in Figure 20b. As noted, a good match was obtained between the experimental and FE load-displacement curves. The softening behavior was efficiently modeled, which proves the precision of the used constitutive models for concrete, steel rebars, and CFRP sheets. As seen in Table 3, this strengthening scheme was predicted to be very efficient at enhancing the ultimate load of wall-like columns. The FE peak axial load of the upgraded specimen SW2 showed increases of almost 87% and 45% over the unstrengthened column CW and the strengthened specimen SW1, respectively.

### 5.4. Strengthened Columns SW3 and SW4

As detailed before, specimen SW3 is the same as SW2 except that two of the three CFRP wrapping layers were bonded to the inside surface of the NSM grooves before the installation of the NSM rebars, and the remaining layer was later bonded to the outer surface of the column. Additionally, specimen SW4 is the same as SW3 except that the 14 ф10 NSM steel rebars were disconnected from the top and bottom bases. As noted from the FE results, the disconnected NSM rebars in specimen SW4 were fully activated during the loading process and continued to share in carrying the axial load until the end of the analysis. Accordingly, the FE results of specimens SW3 and SW4 are identical, as seen in Table 3 and Table 4. Experimental versus FE load-displacement curves for strengthened columns SW3 and SW4 are shown in Figure 20c,d, respectively. As noted, good agreements were obtained between the experimental and predicted load versus displacement curves. The softening behavior was efficiently modeled, which proves the precision of the used constitutive models for concrete, steel rebars, and CFRP sheets. As seen from Table 3, schemes 3 and 4 were predicted to be efficient at enhancing the ultimate load of wall-like columns. The predicted maximum load of specimens SW3 and SW4 showed an increase of almost 56% and 22% over the unstrengthened column CW and the strengthened specimen SW1, respectively. However, schemes 3 and 4 were predicted to be less effective than scheme-2 as the numerically predicted peak loads of specimens SW3 and SW4 were less than specimen SW2 by about 16%, as depicted in Table 3. The reduced efficiency of this scheme compared to scheme-2 is attributed to the less confinement provided to the column section by CFRP wrapping.

**Figure 24:**
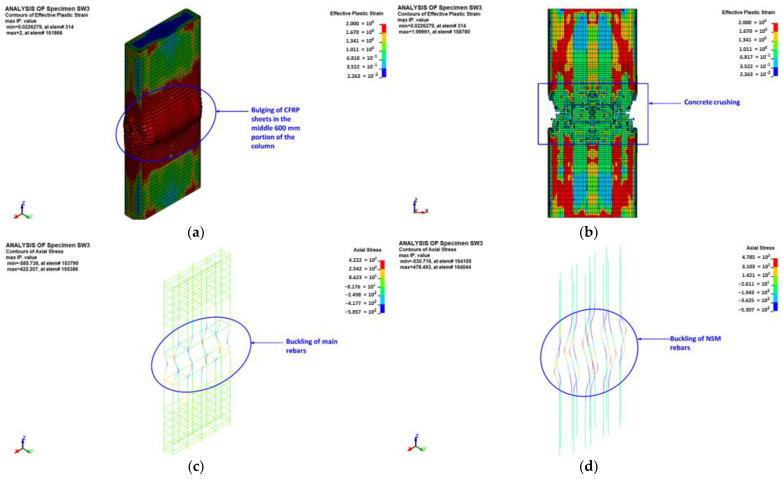

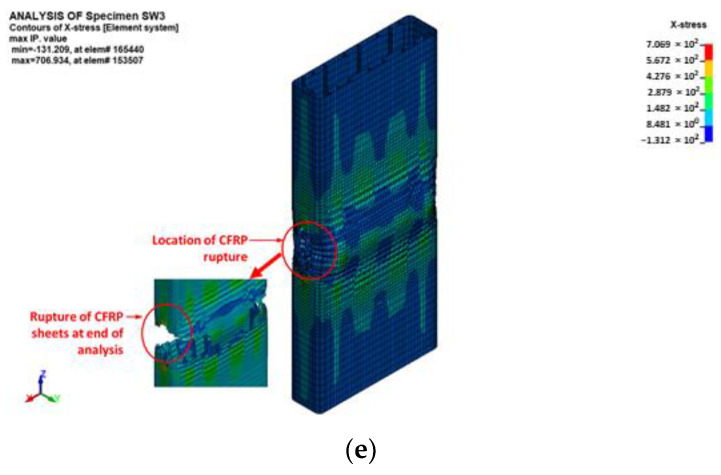
FE mode of failure for strengthened specimens SW3 and SW4: (**a**) bulging of CFRP sheets at peak load; (**b**) effective plastic stress contours for concrete elements; (**c**) axial stress contours for main rebar elements; (**d**) axial stress contours for NSM rebar elements; and (**e**) X-stress contours for shell elements of CFRP sheets.

Figure 24 illustrates the predicted modes of failure for columns SW3 and SW4. As seen in Figure 24, the predicted failure modes agreed well with the experimental results shown earlier in Figure 13c,d. The failure of specimens SW3 and SW4 started with the bulging of the CFRP layers in the middle 600 mm length of the specimen (see Figure 24a). The bulging was concurrent with a sudden drop in the axial capacity of the specimen. The ultimate failure was located in the middle 600 mm portion of the specimen, and it was owing to concrete crushing (Figure 24b) followed by buckling of main and NSM steel rebars (Figure 24c,d). With increased axial displacement, rupture of CFRP laminates occurred in the short side of the column section (Figure 24e). This was confirmed from the predicted peak horizontal strain in the CFRP sheets listed in Table 4, as it was nearly the same as the rupture strain of the CFRP material.

### 5.5. Comparison of Strengthening Schemes

**Figure 25:**
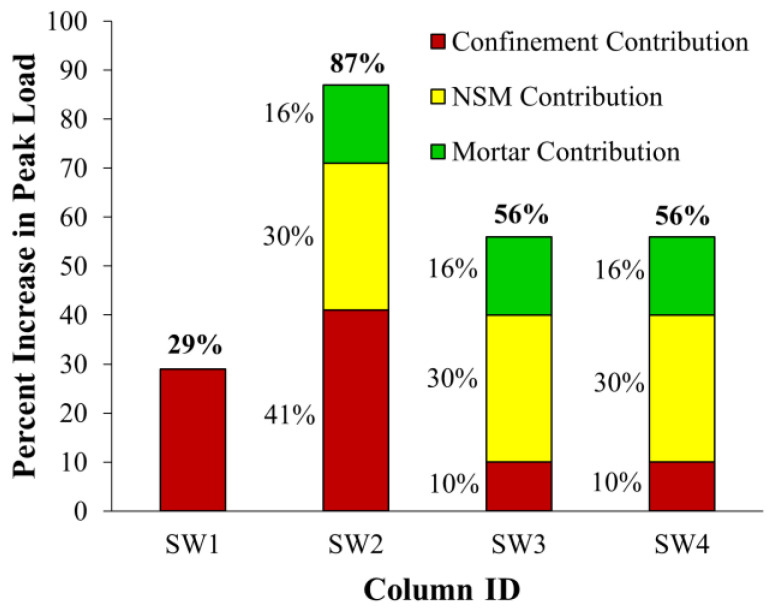
Comparison of strengthened columns with respect to percent enhancement in maximum load (based on FE results).

Based on the FE results of the studied columns, Figure 25 was plotted to illustrate the percent of peak load increase for each strengthening scheme with respect to the control specimen CW. The FE results confirmed that schemes 1 and 2, respectively, provided the lowest and highest peak load increase. Based on the results of the FE modeling, the contribution of each part of the strengthening scheme is also shown in Figure 25. It is generally assumed that the load enhancement due to strengthening is divided into three parts: an increase provided by NSM rebars, an increase due to the increased compressive strength of epoxy mortar, and an increase provided by section confinement. The contribution of each part was approximately calculated from the following equations.

(3) $$\Delta P_{u}=\%\;\mathrm{of}\;\mathrm{peak}\;\mathrm{load}\;\mathrm{increase}\;\mathrm{due}\;\mathrm{to}\;\mathrm{strengthening}=\frac{P_{u}-P_{u-CW}}{P_{u-CW}}\times 100$$

(4) $$\Delta P_{u-NSM}=\%\;\mathrm{of}\;\mathrm{peak}\;\mathrm{load}\;\mathrm{increase}\;\mathrm{due}\;\mathrm{to}\;\mathrm{NSM}\;\mathrm{rebars}=\frac{A_{NSM}f_{y-NSM}}{P_{u-CW}}\times 100$$

(5) $$\Delta P_{u-mortar}=\%\;\mathrm{of}\;\mathrm{peak}\;\mathrm{load}\;\mathrm{increase}\;\mathrm{due}\;\mathrm{to}\;\mathrm{mortar}=\frac{0.85\left(f_{m}^{’}-f_{c}^{’}\right)\left(A_{grooves}-A_{NSM}\right)}{P_{u-CW}}\times 100$$

(6) $$\Delta P_{u-CFRP}=\%\;\mathrm{of}\;\mathrm{peak}\;\mathrm{load}\;\mathrm{increase}\;\mathrm{due}\;\mathrm{to}\;\mathrm{confinement}=\Delta P_{u}-\Delta P_{u-NSM}-\Delta P_{u-mortar}$$

where *P_u_* = peak load of strengthened specimen; *P_u−CW_* = peak load of control specimen CW; *A_grooves_* = cross-sectional area of NSM grooves; and $f_{m}^{’}$ = compressive strength of mortar = 65 MPa.

It should be noted that, in Equations (3)–(6), a linear superposition of the effect of different parts is assumed, and the possible interaction among various components is completely ignored. It is clarified from Figure 25 that in scheme-1 the wrapping of three CFRP layers around the column was predicted to enhance the peak load by about 29%. However, scheme-2 was predicted to increase the peak load by about 87%, and this enhancement ratio was divided into: 30% owing to NSM rebars; 16% due to the increased compressive strength of epoxy mortar compared to the column concrete, and the remaining 41% provided by section confinement. It is noted that in scheme-2, the column section is confined by both CFRP laminates and NSM rebars as both helped in reducing the lateral expansion of the concrete core; however, in scheme-1, the confinement of the column section was only provided by the CFRP laminates. Accordingly, the predicted confinement contribution in SW2 (41%) was larger than that predicted for specimen SW1 (29%). As discussed earlier, connected and disconnected NSM in specimens SW3 and SW4, respectively, provided the same axial load enhancement. Schemes 3 and 4 were predicted to have the same peak load enhancement ratio of 56%. As seen in Figure 25, this enhancement ratio is divided into 30% provided by NSM rebars, 16% owing to the increased strength of epoxy mortar, and the remaining 10% due to section confinement. Compared to schemes 1 and 2, the less confinement provided in schemes 3 and 4 could be owing to the use of one CFRP layer to confine the NSM grooves instead of the three layers used in specimen SW2.

## 6. Conclusions

The core outcomes of this research are summarized as follows:

1. The failure of unstrengthened columns was typical brittle failure caused by the spalling of concrete cover leading to the buckling of main column rebars and consequent total failure owing to concrete crushing.
2. The failure of the strengthened wall-like columns in all the strengthening schemes started with the bulging of the CFRP sheets due to the bulging of the column section and buckling of NSM rebars (if present) and longitudinal column rebars. The ultimate failure of the upgraded columns was through the rupture of CFRP sheets.
3. External wrapping of three CFRP layers in scheme-1 was not effective at enhancing the ultimate load of wall-like columns, as the increase was only moderate by about 27% and 29% for experimental and FE results, respectively. This scheme also had a minor effect on enhancing the secant stiffness of the wall-like column as the increase was limited to 7% and 11% for experimental and FE results, respectively.
4. Using vertical continuous NSM rebars in combination with the wrapping of three CFRP layers onto the exterior column surface (scheme-2) was very efficient at enhancing the axial capacity of the wall-like columns by about 80% and 87% for experimental and numerical results, respectively. Scheme-2 was also very efficient at enhancing the secant stiffness of the wall-like columns by about 45% and 49% for experimental and FE results, respectively.
5. For scheme-3, which was the same as scheme-2, except that two of the three CFRP wrapping layers were bonded to the inside surface of the NSM grooves before the installation of the NSM rebars and the remaining CFRP layer was later attached to the outer surface of the column, the peak load enhancement was about 67% and 56% for experimental and FE results, respectively. However, for scheme-4, which was the same as scheme-3 except that the NSM steel rebars were disconnected from the top and bottom bases, the measured peak load increase was reduced to 57%. Nevertheless, the numerically predicted peak load enhancement for scheme-4 was the same as that for scheme-3 (= 56%). Both schemes were also efficient at increasing the secant stiffness of the unstrengthened column by about 38% to 49%.
6. A good agreement was obtained between the measured and predicted results of tested columns with respect to modes of failure and characteristics of load versus axial displacement for both unstrengthened and strengthened specimens. This demonstrates the precision of the used material models for concrete, steel rebars, and CFRP sheets. Hence, the developed models can be confidently used in future research on upgrading wall-like columns with different parameters such as section aspect ratio, slenderness effect, and different strengthening schemes.

## Figures and Tables

**Table 1:** Matrix of column testing.

Column ID	Strengthening Scheme	No. of Columns
CW	Control specimen (see Figure 1)	2
SW1	This specimen is strengthened using scheme-1 (wrapping of CFRP laminates around the outer column surface) (see Figure 2)	1
SW2	This specimen is strengthened using scheme-2 (wrapping of CFRP laminates around the outer column surface + connected NSM steel rebars) (see Figure 3)	1
SW3	This specimen is strengthened using scheme-3 (bending of two CFRP layers inside the NSM grooves before the installation of NSM rebars and wrapping of the remaining CFRP layers around the outer column surface + connected NSM steel rebars) (see Figure 4)	1
SW4	This specimen is strengthened using scheme-4 (bending of two CFRP layers inside the NSM grooves before the installation of NSM rebars and wrapping of the remaining CFRP layers around the outer column surface + disconnected NSM steel rebars) (see Figure 5)	1
	Total No. of columns =	6

**Table 2:** Properties of constituent materials employed in FEA.

Concrete-Like Materials	Concrete	Epoxy Mortar
Constitutive model	Control specimen	2
Density (kg/m^3^)	2170	2170
Uni-axial compressive strength (MPa)	29.15	65
Poisson’s ratio	0.2	0.2
Maximum size of aggregate (mm)	10	5
**Steel rebars**	**ф8**	**ф10**
Constitutive model	Type 24 (piecewise linear plasticity model)	
Density (kg/m^3^)	7850	
Elastic modulus (GPa)	200	
Poisson’s ratio	0.3	
Yield stress (MPa)	548	531
Tangent modulus (MPa)	86.37	133.75
Plastic strain to failure (%)	9.72	9.73
**CFRP material**		
Constitutive model	Type 54–55 (enhanced comp. damage model)	
Density (kg/m^3^)	1740	
Thickness of single layer (mm)	1.3	
Tensile modulus in long. dir. (GPa)	71.46	
Tensile modulus in transverse dir. (GPa)	3.59	
Longitudinal tensile strength (MPa)	710	
Transverse tensile strength (MPa)	71	

**Table 3:** Comparison of experimental and FE results of load-displacement response for test columns *.

Column ID	Results	*P_y_* (kN)	*P_u_* (kN)	Δ*_s_* (mm)	Δ*_y_* (mm)	Δ*_pu_* (mm)	Δ*_u_* (mm)	*K_s_* (kN/mm)	*E_u_* (kN.mm)
Control specimens									
CW1	EXP	1846	1862	0.27	0.93	1.05	2.01	2810	2866
	FE	1969	1974	0.29	1.04	1.09	2.15	2693	3220
	*EXP/FE*	*0.94*	*0.94*	0.90	*0.89*	*0.96*	*0.93*	1.04	*0.89*
CW2	EXP	1919	2006	0.29	0.93	1.11	1.97	2815	3157
	FE	1969	1974	0.29	1.04	1.09	2.15	2693	3220
	*EXP/FE*	*0.97*	*1.02*	0.97	*0.89*	*1.02*	*0.92*	1.05	*0.98*
Strengthened specimens									
SW1	EXP	2241	2451	0.33	1.02	1.31	2.02	3017	3531
	FE	2439	2540	0.34	1.18	1.39	1.99	2991	3424
	*EXP/FE*	*0.92*	*0.96*	*0.96*	*0.86*	*0.94*	*1.01*	*1.01*	*1.03*
SW2	EXP	3223	3478	0.34	1.06	1.37	1.72	4092	4212
	FE	3491	3686	0.37	1.18	1.40	1.75	4020	4343
	*EXP/FE*	*0.92*	*0.94*	*0.93*	*0.89*	*0.98*	*0.98*	*1.02*	*0.97*
SW3	EXP	3027	3225	0.32	0.92	1.06	1.11	3993	3992
	FE	2971	3088	0.31	0.91	1.10	1.26	4004	3849
	*EXP/FE*	*1.02*	*1.04*	*1.05*	*1.01*	*0.97*	*0.88*	*1.00*	*1.04*
SW4	EXP	2859	3027	0.31	1.00	1.20	1.22	3875	3842
	FE	2971	3088	0.31	0.91	1.10	1.26	4004	3849
	*EXP/FE*	*0.96*	*0.98*	*1.01*	*1.10*	*1.10*	*0.97*	*0.97*	*1.00*

* *P_y_* and Δ*_y_* = load and axial displacement at yielding of longitudinal steel rebars; *P_u_* = peak load; Δ*_s_* = axial displacement at service load level; Δ*_pu_* = axial displacement at peak load; Δ*_u_* = axial displacement at ultimate state; *K_s_* = stiffness at service load level; *E_u_* = energy dissipated at ultimate state; EXP = experimental; FE = finite element.

**Table 4:** Comparison of experimental and FE results of stress-strain relationship for test columns *.

Column ID	Results	$f_{c-avg}^{’}$ (MPa)	$f_{c-act}^{’}$ (MPa)	$\varepsilon_{c-pu}$	$\varepsilon_{cu}$	$\varepsilon_{s-pu}$	$\varepsilon_{s,NSM-pu}$	$\varepsilon_{FRP,u}$
Control specimens								
CW1	EXP	29.79	23.41	0.0026	0.0050	0.0034	-	-
	FE	31.59	25.23	0.0027	0.0054	0.0033	-	-
	*EXP/FE*	*0.94*	*0.93*	*0.96*	*0.93*	*1.04*	*-*	*-*
CW2	EXP	32.09	25.74	0.0028	0.0049	0.0031	-	-
	FE	31.59	25.23	0.0027	0.0054	0.0033	-	-
	*EXP/FE*	*1.02*	*1.02*	*1.02*	*0.92*	*0.95*	*-*	*-*
Strengthened specimens								
SW1	EXP	39.43	33.15	0.0033	0.0050	0.0049	-	−0.0092
	FE	40.65	34.60	0.0035	0.0050	0.0041	-	−0.0098
	*EXP/FE*	*0.97*	*0.96*	*0.94*	*1.01*	*1.19*	*-*	*0.94*
SW2	EXP	55.96	41.11	0.0034	0.0043	0.0038	NA	NA
	FE	58.98	44.56	0.0035	0.0044	0.0038	0.0033	−0.0099
	*EXP/FE*	*0.95*	*0.92*	*0.98*	*0.98*	*1.00*	*-*	*-*
SW3	EXP	51.89	36.92	0.0027	0.0028	0.0034	NA	−0.0099
	FE	49.41	34.63	0.0027	0.0031	0.0039	0.0038	−0.0099
	*EXP/FE*	*1.05*	*1.07*	*0.97*	*0.88*	*0.87*	*-*	*0.99*
SW4	EXP	48.70	33.62	0.0030	0.0031	0.0043	NA	−0.0081
	FE	49.41	34.63	0.0027	0.0031	0.0039	0.0038	−0.0099
	*EXP/FE*	*0.99*	*0.97*	*1.10*	*0.97*	*1.08*	*-*	*0.81*

* $f_{c-avg}^{’}$ = peak average concrete strength; $f_{c-act}^{’}$ = peak actual concrete strength; $\varepsilon_{c-pu}$ = axial concrete strain at peak stress (compression is +ve); $\varepsilon_{cu}$ = ultimate axial concrete strain (compression is +ve); $\varepsilon_{s-pu}$ = axial strain in longitudinal steel rebars at peak load (compression is +ve); $\varepsilon_{s,NSM-pu}$ = axial strain in NSM steel rebars at peak load (compression is +ve); $\varepsilon_{FRP,u}$ = peak horizontal strain in FRP laminates (compression is +ve); $\varepsilon_{sp,u}$ = peak horizontal strain in steel plates (compression is +ve); EXP = experimental; FE = finite element; NA = not available data.

## Data Availability

Not applicable.
